# The impact of rotavirus gastroenteritis on the family

**DOI:** 10.1186/1471-2431-9-11

**Published:** 2009-02-06

**Authors:** T Christopher Mast, Carla DeMuro-Mercon, Claudia M Kelly, Leigh Ellen Floyd, Emmanuel B Walter

**Affiliations:** 1 Merck Research Laboratories, North Wales, PA, USA; 2 Merck Vaccine and Infectious Diseases, North Wales, PA, USA; 3 Duke Clinical Research Institute, Durham, NC, USA

## Abstract

**Background:**

Rotavirus is the leading cause of severe diarrhea in young children and causes substantial morbidity and mortality. Although the clinical aspects have been well described, little information is available regarding the emotional, social, and economic impact of rotavirus gastroenteritis on the family of a sick child. The objectives of this study were to: 1) assess the family impact of rotavirus gastroenteritis through qualitative interviews with parents; 2) compare the clinical severity of rotavirus-positive and negative gastroenteritis; 3) test a questionnaire asking parents to rank the importance of various factors associated with a case of rotavirus gastroenteritis.

**Methods:**

The study enrolled parents and children (2–36 months of age) brought to one of the study sites (outpatient clinic or ER) if the child experienced ≥ 3 watery or looser-than normal stools and/or forceful vomiting within any 24-hour period within the prior 3 days. The clinical severity of each child's illness was rated using a clinical scoring system and stool samples were tested for rotavirus antigen. Parents of rotavirus-positive children were invited to participate in focus group or individual interviews and subsequently completed a questionnaire regarding the impact of their child's illness.

**Results:**

Of 62 enrolled children, 43 stool samples were collected and 63% tested positive for rotavirus. Illness was more severe in children with rotavirus-positive compared to rotavirus-negative gastroenteritis (92% vs. 37.5% rated as moderate/severe). Seventeen parents of rotavirus-positive children participated in the interviews and completed the written questionnaire. Parents were frightened by the severity of vomiting and diarrhea associated with rotavirus gastroenteritis, and noted that family life was impacted in several ways including loss of sleep, missed work, and an inability to complete normal household tasks. They expressed frustration at the lack of a specific medication and the difficulty of treating the illness with oral rehydration solutions, but had a largely positive outlook concerning the prospect of a rotavirus vaccine.

**Conclusion:**

A better understanding of how rotavirus gastroenteritis impacts the family can help healthcare providers ease parental fears and advise them on the characteristics of this illness, practices to prevent infection, and the optimal care of an affected child.

## Background

Since its identification in 1973 [1], group A rotavirus has proven to be the leading cause of severe diarrhea in infants and young children [2]. In the U.S., 2.7 million episodes of rotavirus gastroenteritis occur annually among children <5 years of age, resulting in 410,000 outpatient/office visits, 205,000–272,000 emergency department visits, 55,000–70,000 hospitalizations, 20–60 deaths, and >1 billion dollars of medical care costs [3]. Worldwide, approximately 610,000 deaths annually in children <5 years of age are attributable to rotavirus infection [4].

Traditional measures of childhood diarrheal disease impact (i.e., morbidity, mortality, medical cost) fail to measure the effect illness has on the social and psychological well-being of affected children and their family members – factors the World Health Organization considers to be important aspects of health [5]. Little information is available regarding the impact of rotavirus gastroenteritis on the immediate family of a sick child. Since the disease involves fluid loss associated with severe diarrhea and vomiting, the administration of oral rehydration solutions (ORS) is recommended [6], but parents may be unaware of – or unable to implement – these recommendations. As diarrhea and vomiting persist, parents may call physicians for assistance, schedule medical visits or take their child to the emergency room (ER). The illness associated with rotavirus infection may generate emotional, social, and economic burdens among parents before, during and after contact with the health care system.

Precedents exist for research on the family impact of acute and chronic illness in children. McKenna and Hunt developed a 25-item "Family Disruption Index" after interviewing parents whose children had chickenpox [7], while others have assessed the family impact of caring for a child afflicted with congenital heart disease [8] or atopic dermatitis [9]. Stein and Jessop developed the "Impact on Family Scale" to assess social and psychological impacts of chronic disorders in childhood [10]. However, we are not aware of any studies to assess the family impact of diarrheal disease.

This paper reports the results of an observational study on the family impact of rotavirus gastroenteritis based on focus group and individual interviews with parents. It also presents information regarding the clinical severity of illness associated with rotavirus-positive and negative gastroenteritis, and results from a questionnaire asking parents to rank the importance of various factors associated with their child's illness.

## Methods

The study, initiated during February and March of 2004, was conducted by the Primary Care Research Consortium of the Duke Clinical Research Institute in Durham, North Carolina (Duke Children's Primary Care [3 locations], Durham Pediatrics [2 locations], and Duke Children's Hospital) under a protocol developed within the Epidemiology Department of the Merck Research Laboratories, North Wales, PA. The protocol and study consent form were reviewed and approved by the Institutional Review Board of the Duke University Health System.

### Participants

The study enrolled parents and children (2–36 months of age) brought to one of the study sites (outpatient clinic or ER) if the child experienced an episode of acute gastroenteritis within the prior 3 days. An episode was defined as having ≥ 3 watery or looser-than normal stools within a 24-hour periods and/or ≥ 1 episodes of forceful vomiting. Parents had to be literate in English, give written consent, and be willing to participate in a subsequently scheduled focus group or individual discussion.

### Laboratory and clinical measurements

Whenever possible, a stool sample was obtained from the child at enrollment or was subsequently delivered to study personnel by the parent. Samples were tested for rotavirus antigen at the Duke University Medical Center Laboratory using the ImmunoCard STAT!^® ^Rotavirus immunoassay (Meridian Bioscience, Inc., Cincinnati, OH).

At the time of enrollment, the clinical severity of gastroenteritis was assessed by the physician or nurse health worker based on a direct examination plus parental information and was scored numerically based on a system described in 1988 by Clark et al [11]. This 24-point clinical scoring system was used in pivotal rotavirus vaccine trials and was modified in this cross-sectional study in order to assess clinical severity at a single patient visit. Values of 1–3 (mild, moderate, or severe) were assigned to body temperature (rectal equivalent) and the frequency and duration of diarrhea, vomiting and behavioral symptoms (i.e., irritability, lethargy, seizure). Illness with a clinical severity score ≤ 7 was classified as mild, while scores of 8–14 and 15–21 signified moderate and severe illness, respectively.

### Interviews

Parents of children with a laboratory confirmed case of rotavirus-positive gastroenteritis were invited to participate in either focus group or individual interviews. At the interview session, the interviewer used a semi-structured guide to conduct an open discussion soliciting parental thoughts regarding: 1) severity of the illness, 2) transmission of rotavirus, 3) emotions, 4) schedule disruption, 5) medical care, 6) economic impact, and 7) the prospect of a rotavirus vaccine.

### Pilot quantitative questionnaire

After the interview, parents were given a questionnaire similar to one described by McKenna and Hunt [7], but modified to capture concepts related to rotavirus gastroenteritis. This instrument posed 37 questions regarding feelings, actions, and impacts possibly associated with their child's illness. Parents indicated what they felt were the most significant items and rated their importance. The item impact was determined from the proportion of patients who identified it as important, and the mean importance score [ranging from 1 (least) to 5 (greatest)] attributed to this item (impact score = frequency cited × importance).

### Statistics

Quantitative data were analyzed using Statistical Analysis Software (SAS Institute, Cary, NC, USA), with mean values for continuous variables compared using Student's *t*-test and differences between proportions assessed using either the chi-square test or Fisher's exact test. Relationships between continuous variables were examined by simple linear regression. The level of statistical significance for all tests was set at 0.05.

## Results

### Demographic characteristics of children and parents in the study

Sixty-two children were enrolled in the study between February 2 and March 22 of 2004, a typical rotavirus period in North Carolina [12]. Demographic data were available for 59 of the children; 10 (17%) were enrolled through the ER, while 49 (83%) were enrolled as outpatients. The characteristics of these children and their parents are summarized in Table 1. The children's mean age was 15.3 months and 53% were male. The responding parents had a mean age of 29.8 years and were predominantly female (93%) with at least some college education (66%).

**Table 1:** Demographic characteristics of children and parents participating in the study.

**Children (N = 59)**		
**Characteristic**	**Category**	**N (%)**

**Age, in months**	Mean and (range)	15.3 (3–32.3)
**Age, by category**		
	< 6 months	4 (7%)
	6 – 12 months	20 (36%)
	13 – 24 months	21 (38%)
	> 24 months	11 (20%)
**Gender**	Female	28 (47%)
	Male	31 (53%)

Parents (N = 57*)		

**Age, in years**	Mean and (range)	29.8 (16.8 – 44.7)
**Gender**		
	Female	53 (93%)
	Male	4 (7%)
**Ethnicity**		
	African American	30 (53%)
	Caucasian	17 (30%)
	Other	10 (17%)
**Education**		
	High School or less	19 (33%)
	Some College/College Graduate	29 (51%)
	Graduate/Professional	9 (15%)
**Marital Status **(N = 58)		
	Married	34 (61%)
	Never Married/Separated/Divorced	22 (39%)
**Household Income**		
	Less than $50,000	30 (58.8%)
	$50,000 or greater	21 (41.2%)

* Note: If more than one child per parent was enrolled, only one record was included in the parental analysis.

### Clinical severity of gastroenteritis and rotavirus test results

A stool sample was obtained from 43 (69%) of the children. Lack of a stool sample was more likely if the parent was younger (mean age 26.5 years vs. 31.5 years p = 0.002), unmarried (p = 0.005), or had an annual household income <$50,000 (p = 0.0028).

Twenty-seven (63%) of the 43 stool samples were rotavirus-positive. Emergency room subjects were more likely to test positive than outpatients (100% vs. 53%, p = 0.03). The mean ages of rotavirus-positive and rotavirus-negative children were similar (16.5 months vs. 15.4 months; p = 0.69).

The average clinical severity score for children enrolled in the study was 9.4. However, the score varied considerably by rotavirus status; rotavirus-positive children had more severe scores than negative children (10.64 vs. 7.25; p = 0.0016) and 92% vs. 37.5% were classified with moderate/severe illness, respectively (Table 2).

**Table 2:** Clinical severity of gastroenteritis in children with a stool sample testing positive or negative for rotavirus antigen (n = 43).

	**Number (%)**			
	
**Rotavirus Antigen Test**	All Subjects Tested*	**Mild Illness**	**Moderate/Severe Illness**	**Mean Clinical Severity Score**
Positive	27 (63%)	2 (8.3%)	23 (92%)**	10.64^†^
Negative	16 (37%)	10 (62.5%)	6 (37.5%)**	7.25^†^

**Total**	43	12 (29%)	29 (71%)	

*Although stool samples were obtained from 43 children, severity scores were not available for 2 of the 27 rotavirus-positive children.

**Children testing positive for rotavirus antigen were more likely to have a clinical severity score of moderate-severe than those testing negative (p = 0.0003 by Fisher's exact test).

^†^Children testing positive for rotavirus antigen had a significantly higher mean clinical severity score than those testing negative (p = 0.0016 by Student's *t*-test)

### Qualitative interviews

Seventeen parents (including both parents of one child) of rotavirus-positive children participated in either one of 3 focus group discussions or the 4 individual in-depth interviews conducted between March, 2004 and August, 2005. Summaries of the discussion themes follow and illustrative quotes made by the parents are shown in Table 3.

**Table 3:** Focus group and individual interview themes and representative quotes.

**Theme**	**Illustrative Quotes**
**Illness Severity**	"...and after church we got home, I fed him lunch, and he threw up from then until Wednesday. He couldn't hold anything down. He got so dehydrated he [husband] had to bring him in [to the ER]."
	
	"He was just gagging and vomiting. So we were getting really concerned because he had only one wet diaper in that whole 24 hour period and he couldn't keep the ice chips down."
	
	"Poopy diapers about every 10 minutes...vomiting...had a little bit of fever."
	
	"[M]y husband took off work to stay with her at home, and she had two loose BMs and they were starting to get to watery... She had 11 loose stools that day. So her, I think her biggest part of this was the diarrhea and they were back to back, like 5:26, 5:40, 5:48..."
	
	"Horrible. The vomiting wasn't that bad, because I can deal with that, but the diarrhea was awful."
	
	"..we never had any vomiting.....she had a slight fever,....that night [next day] she had a really loose stool and a fever again.....it's continued [a week later], it's never stopped [the diarrhea].....at least 10 [number of diapers changed daily]."

**Transmission**	"Lysol......extra cleaning......I changed my bed probably every day because she was pooping on it.....And then everything she would touch.....I would wash her hands...".
	
	"We had to kind of partition the house into the sick zone and the healthy zone"
	
	"Somebody would take care of the sick kid and somebody would take care of the healthy...to prevent it from spreading"
	
	".....you just try to do your best and sanitize everything.......with Lysol and spray down the couch and carpet. Clorox wiped off the countertop, I would spray off the door knobs, the bathrooms and I would open all the windows in the house to get fresh air in...My hands were extremely dry and raw from washing, but I knew I had to. I didn't have a choice"
	
	"Is it airborne or what? I'm thinking it's airborne because the baby is passing it around."
	
	"I got it the day after she got sick........I woke up with severe stomach pains.......had to go to Urgent Care.....get a shot ...to stop me from throwing up."

**Emotions**	Oh, man, I was horrified. I guess it's because...I know when the kidneys shut down. That was my main concern; he wasn't drinking anything, eating anything..."
	
	"My husband was scared. He said you got to take her to the doctor now."
	
	"And you're sitting there worried, are you doing the right thing?"
	
	"Very, very anxious because he was so lethargic...it was very scary as a parent."
	
	"The fever was much more elevated.........we were alarmed. We didn't know what she was going through."
	
	"Worried about him getting dehydrated..... I was really upset because I've never seen....he just looked so pitiful vomiting."
	
	"I was upset because seeing his little face, you didn't know what was going on with him and seeing the panic on his face. What's going on? Fix this mommy."

#### Illness severity

Parental descriptions of their child's illness varied, but most felt that rotavirus gastroenteritis was worse than other common childhood diseases. Symptoms ranged in duration from a few days to more than 2 weeks. Most cases started with repeated episodes of vomiting followed by multiple rounds of diarrhea. Some children were reported to have a transient, low grade fever, but several sustained a high fever and in one case this was the initial presenting sign of illness. Repeated vomiting and diarrhea caused the children to become dehydrated and, in some cases, required the administration of IV fluids either in the clinic or the ER.

#### Transmission

Parents had little prior knowledge of rotavirus, and some were uncertain how it was transmitted. However, most believed that good hygiene in the form of frequent handwashing, laundering of clothing and bedding, and the disinfection of objects possibly contaminated with vomit or feces was essential to preventing the infection of other persons in the household. They also discussed the need to keep infected children out of daycare for at least a day after the last episode of diarrhea.

#### Emotions

Parents were frightened by the severity of their child's illness, especially the threat of dehydration from repeated vomiting and diarrhea. They were stressed, fatigued, and frustrated because they were not able to be more effective in relieving the child's distress. Parents were concerned by the behavior of their sick children, who were variously described as whining or crying a great deal and becoming clingy, irritable, and uncharacteristically fatigued or even frighteningly lethargic.

#### Schedule disruption

Many parents missed several days of work, and several stated that caring for their sick child had substantially disrupted their sleep, meal preparation, and the timely completion of other tasks such laundry and housecleaning. Episodes of rotavirus gastroenteritis were sometimes disruptive with respect to social events. One parent said it was necessary to cancel a trip to see family, another noted the illness interfered with plans to attend a birthday party, and a third parent mentioned having to leave a wedding celebration early.

#### Medical care

The primary reason parents sought care for their children was the severity of the illness and concern of its association with a medically serious state of dehydration. Parents were counseled by medical providers to rehydrate the sick child with frequent small volumes of ORS. In some cases this advice was hard to implement because the child either rejected oral fluids or failed to keep them down; several children had to be given IV fluids to combat dehydration and to control vomiting so that oral rehydration could be pursued. Parents were frustrated that there was not more they or the doctor could do to relieve the child's suffering; they felt there should be some specific medication for this illness.

#### Economic impact

Parents mentioned the direct cost of such items as ORS, antipyretics, extra diapers, disinfectants, laundry soap, take-out food (sometimes needed when the child's illness interfered with food preparation at home), and co-payments for a clinic visit to the doctor. They had varied opinions regarding the relative significance of these expenses. While many parents missed several days of work, few felt those absences had much economic impact on the household. Most parents had jobs that continued payment under a variety of policies, such as allowing the employee to take a paid absence against vacation time or maternity leave.

#### Vaccine

The interviews were conducted before the February 2006 licensure of a new rotavirus vaccine in the United States [3]. Parents were asked questions to gauge their interest and attitudes regarding a vaccine that could prevent or attenuate rotavirus gastroenteritis. Parents generally felt that a rotavirus vaccine was needed and would be welcome, especially if their child had a severe course of illness. Opinions were mixed as to whether an oral formulation would be preferable to an injected vaccine. Some favored the oral formulation because it would not increase the large number of injections already given to infants. Others thought children might spit out at least part of an orally administered vaccine, and several expressed the opinion or hope that an injected vaccine might work faster than one given by mouth. Concern over vaccine-related adverse events was expressed by several parents. They wanted to be assured the safety of any rotavirus vaccine would be extensively evaluated and that the benefit of its protective efficacy clearly outweighed any negative side effects.

### Pilot questionnaire on the impact of rotavirus gastroenteritis

Table 4 shows the importance scores assigned to items in the questionnaire. The rankings are largely consistent with comments made during the interviews. Parents expressed a high level of worry over the child's illness and were heavily impacted by the need to attend their child at night, the time spent changing diapers soiled by frequent episodes of diarrhea, and the effort expended in attempting to feed and keep the child adequately hydrated. Parents were also much impressed by how the illness affected the child's behavior, with the need to be cuddled a lot, a loss of appetite, having little energy, and being sleepy all the time ranked as prevalent aspects of this illness. The direct economic impact of this illness, and its effects on social interaction and the completion of household tasks were rated as relatively less important for the family.

**Table 4:** Importance scores based on parental responses to a pilot questionnaire on the relative importance of factors associated with caring for a child with rotavirus gastroenteritis.

**Rank Order**	**Item**	**Rank Mean Score**
1	I was worried about my son/daughter	4.44
2	Wanted to be cuddled a lot	4.24
3	Ate very little	3.93
4	Was sleepy all the time	3.65
5	Did you spend a lot of time changing diapers because of diarrhea?	3.48
6	Did you spend time trying to fee him/her rehydration solution?	3.32
7	Had very little energy	3.32
8	I felt tense	3.31
9	I had to get up during the night to see my son/daughter	3.22
10	I felt very tired	3.21
11	Wanted someone with him all the time	3.20
12	Got tired easily	3.06
13	Was miserable	3.00
14	Whined a lot	2.96
15	Did you spend time trying to keep his/her temperature down?	2.86
16	Was irritable	2.81
17	Did you have to spend time in the house more than you wanted to?	2.37
18	Did not want food	2.34
19	I had to stay home from work	2.24
20	Was moody	2.16
21	Seemed to have lost interest in everything	1.95
22	Did you see family and friends less?	1.87
23	I felt like I had to rush around	1.83
24	Slept badly at night	1.75
25	I had to change plans at the last minute	1.74
26	Did you have to rush to get jobs done?	1.38
27	Did you have to spend most of the day amusing your son/daughter?	1.05
28	Did you get behind in your house cleaning?	1.00
29	Traveling to the doctors office was a strain	0.83
30	I had no time for other family members	0.83
31	Was restless	0.82
32	I couldn't travel out of the city	0.53
33	I felt nobody understood the burden	0.52
34	Did you have to ask someone not to visit you socially	0.48
35	I felt like this was a financial burden	0.32
36	Traveling to the hospital was a strain	0.29
37	I was depressed	0.05

(Rank Mean Score 1 = Not Important to 5 = Very Important)

## Discussion

This is one of the first published studies to assess the family impact of rotavirus gastroenteritis. The research adds to the literature by demonstrating that children affected by rotavirus gastroenteritis are part of families that can be substantially impacted by the illness of a child. The illness results in subsequent disruption to family routine, parental work schedule, and the parents' sense of control. The qualitative research technique of using focus group or individual interviews was intended to provide richer context to the often cited clinical and population level impact of this disease. By design, qualitative health studies are often much smaller than quantitative studies, since they seek in-depth narratives from subjects using their own words to describe disease related factors of importance to them.

Our data suggest that rotavirus gastroenteritis has significant impact on the physical and emotional well-being of the child but also the parent. Many parents expressed fear over the possibility the child might suffer medically significant dehydration, distress regarding the child's discomfort and their inability to rapidly relieve the child, and frustration over problems with administering ORS as well as the disruptive effects of this illness on parental sleep, work, and household routines.

An opinion expressed by some parents that the risk of rotavirus infection is relatively low and has only slight economic impact stands in contrast to the literature and points to the need for better education about this disease. In fact, the cumulative risk of rotavirus infection is nearly 100% by the time children reach 5 years of age [3], and children treated in the ER, who were given IV fluids and blood tests to check electrolytes, and perhaps other procedures, certainly incurred substantial medical costs. However, the qualitative interviews in this study assessed only parental perceptions of relatively minor out of pocket expenditures (e.g., the cost of purchasing extra diapers or ORS) and did not consider other documented costs from the payer perspective.

Although the study was designed primarily to elicit qualitative data, the observation that 63% of stool samples collected in our study during the winter/spring of 2004 were rotavirus-positive complements other quantitative studies showing that this viral infection is an important reason for visits by young children in the U.S. to outpatient clinics or admission to hospitals [13,14]. Our results also accord well with a large hospital-based study showing that rotavirus gastroenteritis is more severe than gastroenteritis caused by other enteric pathogens [15].

The study was also useful as a preliminary test of a questionnaire probing parents' perspectives regarding the importance of various factors involved in caring for a child with rotavirus gastroenteritis. The rank order and numeric scores parents assigned to items on that questionnaire closely reflected the concerns expressed in the focus group and individual interviews, with factors of major importance including parental feelings of fear regarding the severity of the child's illness, concern for the child's suffering and behavioral changes, sleep disruptions and the extra work involved in attending to and trying to keep the child effectively hydrated. This preliminary assessment will be useful in refining the questionnaire for application in future rotavirus burden of illness studies involving larger samples of parents and primary caregivers.

As in every study, there were limitations to our findings. Although 62 subjects were enrolled in the health care setting, stool samples for rotavirus testing could not be obtained for 19 (31%) of the children, and only 17 parents of the 27 rotavirus-positive children ultimately participated in the focus group or in-depth interviews. We attribute the latter difficulty to the problem of scheduling multiple parents to meet at a mutually convenient location, and the likelihood that once a child recovers the parent may be less inclined to continue discussion of the illness. Nevertheless, we obtained a rich qualitative dataset that provides a previously unavailable perspective on rotavirus gastroenteritis. Those parents who participated in the interviews spanned a broad socioeconomic spectrum and so provided a good range of the perspectives of parents who may have to deal with this common childhood illness.

Although this study enrolled a diverse patient population (Table 1), our study was conducted only in the United States. Given the substantial international burden of rotavirus disease in other developed and lesser developed countries [4], further research would be helpful to understand the global impact of rotavirus disease on families. In particular, there are substantially different types of medical care systems and patient access to medical care in international settings that could result in family impact from rotavirus disease that differs from the results reported here. Given the lack of other available data, the research methods utilized in this study could be used to provide additional family impact data from other geographic and cultural settings.

Two rotavirus vaccines are now licensed for pediatric use in the US. The first of these was the pentavalent reassortant rotavirus vaccine, RotaTeq™ [16], from Merck & Co., Inc. and was licensed in February 2006. The public health impact of the introduction of RotaTeq™ in the US was recently documented by a substantially delayed onset and diminished magnitude of rotavirus activity in the 2007–08 season compared with previous years [17]. The second licensed vaccine is the monovalent vaccine licensed in April 2008 (Rotarix™, GlaxoSmithKline Biologicals, Inc.).

Despite the availability of rotavirus vaccines that can effectively prevent severe rotavirus disease, this study highlights that more education about rotavirus disease and vaccination is needed for parents and their health care providers. A study conducted in the US by the US Centers for Disease Control suggested that a general lack of parental familiarity with rotavirus disease, its potentially serious consequences, and the need for preventative vaccination may be a barrier to the acceptance of the new vaccines [18]. Similar results were found in a study conducted among providers in several international settings [19]. Both studies highlighted the need for better dissemination of information about rotavirus disease in order to prioritize the disease and to highlight the benefits of vaccination.

Since parents or providers may not know the etiology of the severe diarrhea that is being caused by rotavirus, further education on the management of children with rotavirus infection is important. The management of diarrhea has typically focused on the use of oral rehydration therapy (ORT), which is recommended for all age groups and for diarrhea of any etiology [6]. All families should be encouraged to have a supply of ORS in the home at all times and to start therapy with a commercially available ORS product as soon as diarrhea begins. However, the severe vomiting that is characteristic of rotavirus disease often makes the successful administration of ORT difficult [19]. Therefore it is important that providers educate parents how to recognize signs of illness or treatment failure that necessitate medical intervention [6].

## Conclusion

A better understanding of how rotavirus gastroenteritis impacts the family, can help healthcare providers ease parental fears and advise them concerning the course of this illness, practices to prevent infection, and the optimal care of an affected child.

## Competing interests

TCM and CMK are employees of Merck & Co., Inc. CD-M was an employee of Merck & Co., Inc. at the time the study was conducted. LEF and EBW received funding from Merck & Co., Inc. to conduct the study. EBW is a member of a pediatric vaccine advisory board and is a speaker for Merck & Co., Inc.

## Authors' contributions

TCM conceived of the study, and participated in its design, coordination and analysis, and helped to draft the manuscript. CD-M participated in the design and coordination of the study, facilitated the focus groups and interviews, and assisted with the analysis of the qualitative interviews. CMK conducted the quantitative analysis and helped to draft the manuscript. LEF participated in patient recruitment and coordination of the study. EBW participated in the design of the study, oversaw patient recruitment and provided clinical oversight and helped to draft the manuscript. All authors read and approved the final manuscript.

## Pre-publication history

The pre-publication history for this paper can be accessed here:


